# A novel real-time ultrasonic method for prion protein detection using plasminogen as a capture molecule

**DOI:** 10.1186/1472-6750-7-43

**Published:** 2007-07-20

**Authors:** Carmen Negredo, Eoin Monks, Torres Sweeney

**Affiliations:** 1 Veterinary Sciences Centre, School of Agriculture, Food Science and Veterinary Medicine, University College Dublin, Belfield, Dublin 4, Repulic of Ireland

## Abstract

**Background:**

High resolution ultrasonography (HR-US) can monitor the molecular changes and biochemical interactions between proteins in real-time. The aim of this study was to use HR-US to characterize the real-time interactions between plasminogen coated beads and PrP^Sc ^and to determine if this approach could be applied to the identification of animals affected by prion diseases. Plasminogen, immobilized to beads, was used as a capturing tool for PrP^Sc ^in brain homogenates from scrapie affected sheep and the binding reaction was monitored in real-time in an ultrasonic cell.

**Results:**

Changes in the ultrasonic parameters suggested that three processes occurred during the incubation: binding, protein-protein network formation and precipitation and that these processes occurred in a concentration dependent manner. Conversely, when homogenates from normal sheep were similarly examined, no evidence for the occurrence of these processes was found indicating the specificity of the interaction between the plasminogen coated beads and PrP^Sc^.

**Conclusion:**

These results indicate firstly, that the plasminogen coated beads binded selectively to PrP^Sc ^and secondly, that a HR-US system can discriminate between scrapie affected and non-affected samples and thus has potential as a tool for the rapid diagnosis for prion diseases. This approach has the significant advantage of not requiring a proteinase K pre-digestion step, which is routinely used in current PrP^Sc ^detection assays.

## Background

Prion diseases such as CJD in humans, BSE in cattle and scrapie in sheep are a group of neurodegenerative disorders, which are characterised by the accumulation in the central nervous system of the protease resistant form (PrP^Sc^) of a host-coded membrane glycoprotein (PrP^c^) [1]. The transformation of PrP^c ^into PrP^Sc ^implies a conformational change from a mainly alpha helical form into a beta sheet rich structure [2,3]. This conformational difference is responsible for the distinct physicochemical properties of both isoforms. While PrP^c ^exist as a monomer and it is rapidly degraded by proteinase K (PK), the infectious isoform PrP^Sc^, forms detergent-insoluble aggregates and displays a higher resistance to degradation with PK [4,5].

Most currently used diagnostic techniques are based on the immunological detection of PrP^Sc ^using antibodies specific to the prion protein. As antibodies used on current validated assays are not able to differentiate between PrP^Sc ^and PrP^c^, these diagnostic procedures rely on the elimination of PrP^c ^by PK digestion, PrP^Sc ^remaining due to its PK resistance. However, the use of PK sample pre-treatments may compromise the sensitivity of an assay as some PrP^Sc ^conformations are known to be relatively protease-sensitive.

Many recent investigations are focused on increasing the sensitivity of current diagnosis assays and much effort has been directed toward the development of novel reagents that could be used as sensitive tools for prion detection. Several such reagents, aimed at the specific detection of PrP^Sc^, include nucleic acid-aptamers [6], anti-DNA antibodies [7], PrP^Sc ^specific monoclonal antibodies antibodies [8,9] and plasminogen protein [10].

Plasminogen, a plasma proenzyme involved in the process of fibrinolysis [11], has shown potential for the detection and differentiation of PrP^Sc ^without the necessity for a PK pre-treatment. The specific binding of plasminogen, covalently bound to magnetic beads, to PrP^Sc ^aggregates in tissues from several species has been demonstrated [10,12]. While binding of PrP^c ^may occur, PrP^Sc ^is preferentially bound under experimental conditions that enhance the formation of fibrils [13].

High-resolution ultrasonic spectroscopy is a novel real-time analytical non-destructive technique for material analysis, in which changes in the behaviour of ultrasonic waves, as they pass through a test material, may provide information regarding the structure and composition of the material. Ultrasonic spectroscopy allows simultaneous measurements of two independent parameters: velocity and attenuation, both of which are very sensitive to changes in intermolecular interactions and molecular organization. Attenuation is a measure of the energy loss in the compressions and decompressions produced as an ultrasonic wave passes through a material and gives information on the microscopic structural organisation of the material. Ultrasonic velocity is a measurement of the density and elastic response of the material to the oscillating pressure and provides information regarding its molecular organization. The ability of an ultrasonic system to monitor both structural and chemical processes in real time has been demonstrated. For example, it has been shown to be well suited to a wide range of applications such as the study of aggregation and gelation in food colloids, conformational transitions in polymers and biopolymers, and phase transitions and formation of micelles [14-18]. It has also been recently applied to the analyses of chemical reactions [19,20]. Advantages of this technique include the ability of the ultrasonic wave to propagate through a broad range of samples, including opaque materials (in contrast to optical spectroscopy), and the opportunity to carry out on line analysis because ultrasonic detection can be done without pre-treatment of the material. Another distinct advantage is the high resolution in measurements of velocity, allowing detection of concentrations down to ppm level.

A combination of specific reagents for the disease associated prion protein and this novel ultrasonic technique make this approach extremely promising for the analysis and diagnosis of prion disease. Thus, the aim of this study was to investigate the interactions between plasminogen coated beads and PrP^Sc ^in scrapie affected brain tissue, particularly in the absence of a proteinase K pre-digestion step, with a view to exploring its potential for use as a sensitive detection system for PrP^Sc^.

## Results

The interaction of PrP^Sc ^to plasminogen was analyzed in real time using the ultrasonic spectroscopy measurements of velocity and attenuation. In each experiment, the evolution of ultrasonic profiles was investigated over a period of approximately four hours after the addition of a plasminogen coated bead solution (10 μl added giving a final concentration of 2 × 10^7^) or alternatively in the absence of beads. Specificity was demonstrated using uncoated beads. In a first series of experiments, binding kinetics were analyzed by titration of PK undigested homogenates at three concentrations 10, 5 and 2%. As the 2% homogenate gave the clearest results, all further experimentation focused on this concentration and only these results are reported here. Similarly, velocity measurements gave the most valuable information; attenuation measurements failed clearly to differentiate different homogenate variables. Thus, only the results of the velocity measurements are presented here.

### PK untreated homogenates

**Figure 1:**
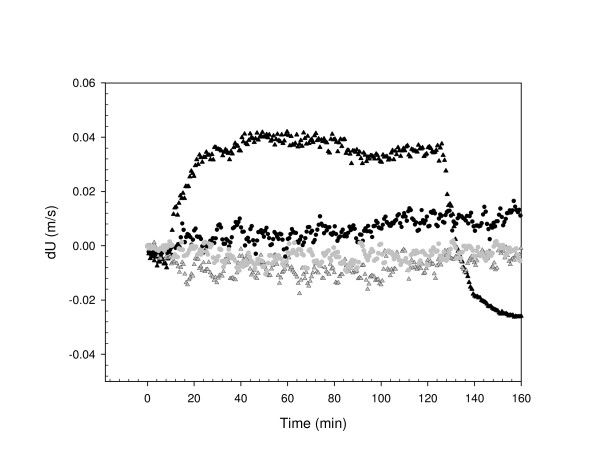
Illustration of ultrasonic velocity (dU) profiles of PK-untreated scrapie infected and healthy brain homogenates prepared at 2% in the presence and absence of plasminogen coated beads. Binding reaction was performed in the reaction buffer at 25°C. A 10 ul-buffer solution with coated beads (2 × 10^7^) or without (0) was added to each sample (t = 0). Ultrasonic profiles of scrapie infected homogenate in the presence (black triangle) and absence (grey triangle) of beads and healthy brain homogenates in the presence (black circle) and absence of beads (grey circle) are shown over approximately 3-hour of the reaction.

In scrapie affected, PK untreated homogenates, velocity profiles demonstrated three distinct stages during the incubation period (see Figure 1). The first stage is characterized by an increase in the velocity values, reaching a peak after about 40 minutes. The second stage was characterized by an extended plateau period, lasting approximately 90 minutes and finally there was a sharp decrease, taking about 10 to 20 minutes to reach T0 levels. This pattern of binding was found consistently in repeat experiments.

By contrast, scrapie unaffected homogenates gave profiles (Figure 1) which were quantitatively and qualitatively distinct from those of affected samples (P < 0.05). They showed a gradual but small increase over the four hour period of study and were very similar to those of the control samples, both scrapie affected and unaffected, which were performed in the absence of beads (Figure 1).

Thus, it was only in the scrapie affected tissues that there evidence of a significant reaction and these results argue for an interaction between the plasminogen coated beads and PrP^Sc ^but not between the plasminogen coated beads and PrP^c ^or any non-PrP component of the sample.

### Effect of PK

Further studies to investigate the specificity of the velocity profiles were performed using PK-treated homogenates. By contrast to the three-phase velocity profile seen with PK untreated scrapie positive tissues, PK appears to have the effect of eliminating the plateau phase giving instead, a two-phase reaction. This comprised firstly a gradual increase over a period of ~70 minutes and followed by a sudden decrease, taking ~10 minutes to reach T0. The amplitude of the Phase-1 increase in these PK treated tissues was always less than that seen in the PK untreated.

Velocity profiles in scrapie unaffected tissues showed no evidence of any reaction and no significant difference was seen between those which were PK treated and those untreated (P > 0.05).

## Discussion

The ability of plasminogen coated beads to selectively bind PrP^Sc ^was confirmed in experiments involving PK-treated healthy and PK-treated infected brain samples. The results of the PK-treated healthy samples, which showed no evidence of any binding, indicated that no non-PrP molecule is bound by the plasminogen coated beads. Thus, in PK treated homogenates, only PrP^Sc ^is bound to plasminogen. In experiments involving PK untreated homogenates, we have demonstrated that no binding occurs in healthy samples, indicating that PrP^c ^does not bind in these reaction conditions. By contrast, in PK untreated scrapie affected samples, there is good evidence that PrP^Sc ^is bound to plasminogen. These results corroborate previous investigations, which indicated a selective binding response of plasminogen to PrP^Sc ^[10,12] demonstrating the feasibility of plasminogen and the potential of this technique to be used as a diagnostic tool to differentiate between PrP isoforms.

Controversial to these results are the studies of Ellis *et al*. [21], Ryou *et al*. [22], Kornblatt *et al*. [23], and Cuccioloni *et al*. [24]. These investigations, performed with recombinant prion protein as well as purified full-length PrP^c^, suggested that PrP^c ^could also interact with plasminogen. The apparently contradictory evidence might be explained by the different binding properties of plasminogen kringle domains to PrP in varying detergent conditions. It has been suggested that the preferential binding of plasminogen to one PrP isoform or another depends of the physical state of PrP in the particular detergent solution [12,22]. Fischer *et al*. [10] and Maissen *et al*. [12] pointed out that the high amyloid content of PrP^Sc ^and/or its aggregated state could have favored the binding of plasminogen to the PrP^Sc ^isoform over the soluble PrP^c ^form. Thus, the binding of prion isoforms to plasminogen is affected by the detergent extraction conditions. Shaked *et al*. [13] reported that the selective binding of plasminogen to PrP^Sc ^occurs under detergent conditions that cause raft disruption and enhance the aggregate state of PrP^Sc^. These authors reported that PrP^c^, extracted from natural sources, was able to interact to plasminogen only in detergent conditions in which rafts remained intact. In the present study, homogenates were prepared in Sarkosyl, an anionic detergent which promotes aggregation, and most likely, solubilises rafts [25,26]. It is therefore likely that the reaction conditions used in this study promoted the interaction of plasminogen and the aggregated PrP^Sc ^isoform. There was no evidence that endogenous palsminogen would interfere with this assay in this study as all five scrapie positive tissues interacted with the plasminogen coated beads.

Although velocity profiles of both PK-untreated and treated scrapie infected samples revealed binding, complex formation and precipitation, the magnitude of velocity changes, rate of binding and time intervals at which precipitation occurs varied between samples. Conclusions could not be drawn in accordance with the quantitative kinetics of rate of binding in those curves as the accurate concentration of protein and therefore the number of binding sites on the surface is unknown. However, comparative qualitatively analyses of velocity profiles of PK-treated and untreated samples revealed that the rate of binding and magnitude of change in velocity was higher and faster in PK-untreated samples than PK-treated samples. In contrast, the precipitation of protein complexes seems to occur more rapidly in PK-treated than in PK-untreated samples. These results suggest a differential binding response of plasminogen to PK treated and untreated PrP aggregates. Variations in velocity parameters observed could be explained by the formation of two different types of aggregates possibly very similar in size but different in the structure and the amount of accessible groups at the surface of the aggregates. Plasminogen is a blood serum protein, which has been shown to bind to different molecular surfacesthat contain exposed carboxy-terminal lysines [11,27]. This interaction is kringle mediated. The mechanisms of binding of PrP^Sc ^to plasminogen are unclear and investigations in this process have been curtailed by the lack of structural information on the PrP^Sc ^prion protein. However, it has been demonstrated that the interaction of plasminogen to PrP^Sc ^aggregates is lysine mediated [10]. Sheep prion protein contains several clusters of lysine residues in the core (104, 107, 109, 113, 188, 197, and 207) and also in the flexible tail (25, 26, and 29). The N-terminal core of the protein is a flexible extended tail, thus it is very sensitive and susceptible to the degradation by PK regardless the source of PrP^Sc ^[28]. It is possible that the treatment of PrP^Sc ^aggregates with proteinase K not only may remove important lysine binding sites but induce a conformational change leading to molecular structural rearrangement that might masks binding sites and other parts of the PrP molecule. New structural rearrangement of aggregates could induce a decrease in the ligand-binding efficiency of plasminogen kringles. Proteins with carboxy-terminal lysyl residues appear to function as plasminogen binding sites [27]. However, it is known that other structural characteristics of peptides and proteins, such the presence of additional lysyl residues proximal to the aminus terminus, can increase their affinity for plasminogen [27]. Several studies performed with recombinant prion protein have demonstrated the importance of n-terminal sequences in enhancing plasminogen binding activity [22,29].

If two different structural types of aggregates exist, with the PK-untreated type having a higher affinity for plasminogen than the PK-treated type, then, differences in velocity changes between PK-treated and untreated samples may be expected. Contributions to velocity changes depend on the elastic properties of the newly protein-protein network and therefore on the number of involved interactions between aggregate particles. In the case of PK-untreated samples the protein network is rapidly formed and stabilized by lysine interacting plasminogen kringles. In contrast, PK-treated aggregates which are not able to form additional bonds interact slowly with plasminogen forming protein-protein complex of reduced stability. The faster rate of binding on PK-untreated samples compared to PK-treated could be explained in terms of binding cooperativity. Ryou *et al*. [22], showed a positive cooperative binding process between kringle domains and recombinant PrP.

Changes in ultrasonic velocity and attenuation observed during the course of the experiments suggest that considerable physicochemical alterations were occurring in the solution. Ultrasonic velocity is determined by the density and elastic response of the medium to the oscillating pressure and this parameter can be expressed in terms of compressibility [30]. An increase on ultrasonic velocity at initial stages of the reaction suggests that both conformational changes and protein-protein complex formation processes took place during the binding event. Both, chemical interactions, involved in the binding process of plasminogen to PrP^Sc^, along with structural characteristics of the newly formed protein network, could have contributed to the positive velocity changes observed. Conformational changes in the plasminogen protein molecule upon binding possibly contributed to the increment observed in the velocity values by decreasing the compressibility of the system. Significant conformational changes involving changes from a compact structure to a less compact in binding to small molecules such as aminocarboxylic acids or proteins such as fibrin have been frequently reported [31-33]. A change from a closed to an open conformation has been reported to occur, upon binding of plasminogen to a ligand. It has been suggested that this conformational change is able to produce a decrease in the system volume possibly due to the increase in exposure of surface area to the solvent, disruption of electrostatic interactions and decrease of size voids [34]. The increase in exposure of the protein to water molecules (i.e. hydration) has a significant effect on the ultrasonic velocity. An increase of water in the hydration shell of the solute molecules results in a rise in ultrasonic velocity in solutions due to lowering in the compressibility of the overall system. In the present study, similar factors contributing to a decrease in the compressibility of the system were expected to occur upon this type of conformational change. The increase in velocity is consistent with the protein changing from a compact to a more loosening structure. It was interesting that the most consistent profiles were detected at the most dilute concentration of homogenate (2%). As proteins change in conformation, cavities in the interior of the molecule are created and a rearrangement of atomic groups take place. Groups that were initially buried within the molecule may become exposed during the conformational transformation inducing variations in the hydrogen bonding and hydrophobic interactions of the protein. Positive velocity changes could also be related to the changes on elasticity in the solution due to the formation of the protein-protein network. Elasticity can been used to characterize protein network formation. Formation of a protein network introduces resistance to deformation and elasticity. As the protein-protein network is formed, the medium become more rigid and compact, so the velocity of the sound will be expected to increase in this new less elastic medium. Eventually, this protein interacting network precipitates restoring the initial medium properties. Precipitation was indicated by a decrease in velocity values. It is probable that the reaction conditions in the 2% homogenate was most favourable to detecting these molecular interactions.

## Conclusion

this study highlights the potential of the HR-US system to detect plasminogen-PrP^sc ^aggregates and to discriminate between PrP isoforms. The specificity of the interaction of plasminogen to PrP^Sc ^was demonstrated on scrapie PK-untreated and PK-treated samples and confirmed on healthy brain samples. This test is significant as it does not require a proteinase-K pretreatment step, which considerably improves the specificity of the assay.

## Methods

### Principles of plasminogen capture ultrasonic assay

In this study, the ultrasonic system was used to continuously monitor the binding reaction of plasminogen to sheep PrP^Sc ^with the aim of differentiating scrapie affected from non-affected brain tissues. Various preparations from normal or scrapie-affected brains were prepared, each at three different homogenate concentrations (2, 5, and 10%) and incubated with plasminogen coated beads in an HR-US cell. Incubation conditions were optimised to promote preferential binding of PrP^Sc ^to plasminogen (J. Grassi, personal communication). Both PK digested and undigested homogenates were tested in order firstly to investigate the specificity of PrP^Sc ^binding in the presence of PrP^c ^and secondly, to investigate the possibility that a digestion step might be capable of being omitted – a great advantage should a TSE diagnostic assay be developed.

### Experimental procedures

#### Covalent coupling of plasminogen to magnetic beads

Coupling of plasminogen to magnetic beads was performed following manufacturer's instructions. Briefly, 100 μg of human plasminogen (Sigma) was covalently bound to paramagnetic Tosyl-activated beads (M-280 Dynabeads, 2 ml) purchased from Dynal (Oslo) in 1 ml of coupling buffer (0.1 M borate pH 9.5). After incubation for 48 hours at 4°C, the beads were washed twice with phosphate buffered saline (PBS), containing 0.1% (w/v) BSA, for 5 min. Blocking of free tosyl-groups was accomplished by incubating the beads with 0.2 M Tris pH 8.5 containing 0.1% (w/v) BSA for 24 h at 20°C. Finally, beads were washed once in PBS pH 7.4 containing 0.1% (w/v) BSA for 5 min at 4°C and made up to a final concentration 2 × 10^9^/ml in PBS/BSA buffer containing 0.02% sodium azide. The coated bead suspension was stored at 4°C in.

#### Brain tissues and homogenate sample preparation

Brain tissues (cerebellum) were kindly supplied by the CVRL (Abbotstown). Scrapie affected tissues were from naturally infected sheep. Animals (two male and 3 female) were aged between 2–4 years of age Samples were collected at post-mortem and scrapie diagnosed by histopathology and confirmed by immunohistochemistry. Homogenates (20% w/v in 5% sucrose) of brains from five normal and five scrapie infected sheep were prepared and aliquots were stored at -20°C. Duplicate aliquots were brought to 2, 5 or 10% by dilution with ice-cold capture buffer (0.1 M phosphate buffer pH 7.4, 0.5% M NaCl, 0.1% Sarkosyl) with or without PK (Sigma). Samples digested with PK were incubated with 5 μg/ml at 37°C for 20 min. The digestion was terminated by addition of Pefabloc (protease inhibitor purchased from Sigma) to a final concentration of 4 mM. Pefabloc at the same final concentration was also added to the undigested brain samples.

#### High-resolution ultrasonic spectroscopy analyses

Ultrasonic velocity and attenuation were measured using a HR-US 102 ultrasonic spectrometer from Ultrasonic Scientific Ltd.; cell temperature (25°C) was controlled with a Haake F8 water circulator bath (Haake C5) (Karlsruhe, Germany). Measurements were taken at different frequencies, 5000, 8000 and 11400 MHz, the margin of error being 0.2 mm/s and 0.1% for velocity and attenuation respectively. All measurements were carried out in a differential mode with two identical acoustical resonator cells: a measuring and a reference cell. Solutions were degassed prior to loading into the resonance cells. A volume of 900 μl of brain homogenate, prepared in capture buffer, was added to the measuring cell and the reference cell was filled with the same volume of capture buffer only. Prior to ultrasonic measurements the samples were allowed to equilibrate to the desired temperature (25°C) for approximately 20 minutes. Each reaction was initiated by addition of a solution of plasminogen coated beads in a single 10-μl aliquot (2 × 10^7 ^beads) to both measuring and reference cells, the solution being delivered with a Hamilton syringe by injection through a small hole in the lid of each cell. Reaction mixtures were kept constantly stirred at 700 rpm. Changes in velocity and attenuation in both cells were continuously monitored for 3–4 hours and the difference between measuring and reference cells was automatically calculated and recorded. Control experiments without beads were performed under the same conditions as described above, a 10-μl aliquot of capture buffer rather than beads being injected into the measuring and reference cell. All experiments were performed in duplicate or triplicate. Data were analysed using a general linear model repeated measures analysis of variance.

## Authors' contributions

CN generated and analysed all of the experimental data and contributed to writing the manuscript. EM assisted with the prion protein handling and methodologies and contributed to writing the manuscript. TS had overall responsibility for supervising the project, interpretation of the data and contributed to writing the manuscript.

**Figure 2:**
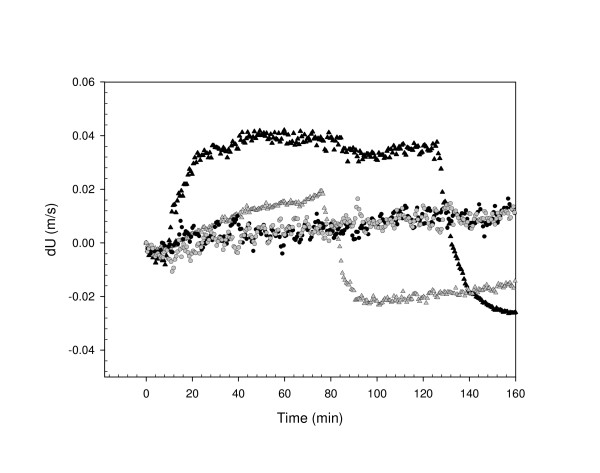
Illustration of ultrasonic velocity (dU) profiles of PK-untreated and PK-treated scrapie infected and healthy brain homogenates prepared at 2%. Binding reaction was performed in the reaction buffer at 25°C. A 10 ul-buffer solution containing plasminogen coated beads (2 × 10^7^) was added to each sample (t = 0). Scrapie PK-untreated (black triangle), PK-treated (grey triangle) and healthy PK-untreated (black circle) and PK-treated (grey circle) are shown over approximately 3-hour of the reaction.
